# Supplementary figures and images for: Inferring directional relationships in microbial communities using signed Bayesian networks
Source: BMC Genomics. 2020 Dec 21;21(Suppl 6):663. doi: 10.1186/s12864-020-07065-0 (PMC7751116; doi:10.1186/s12864-020-07065-0)

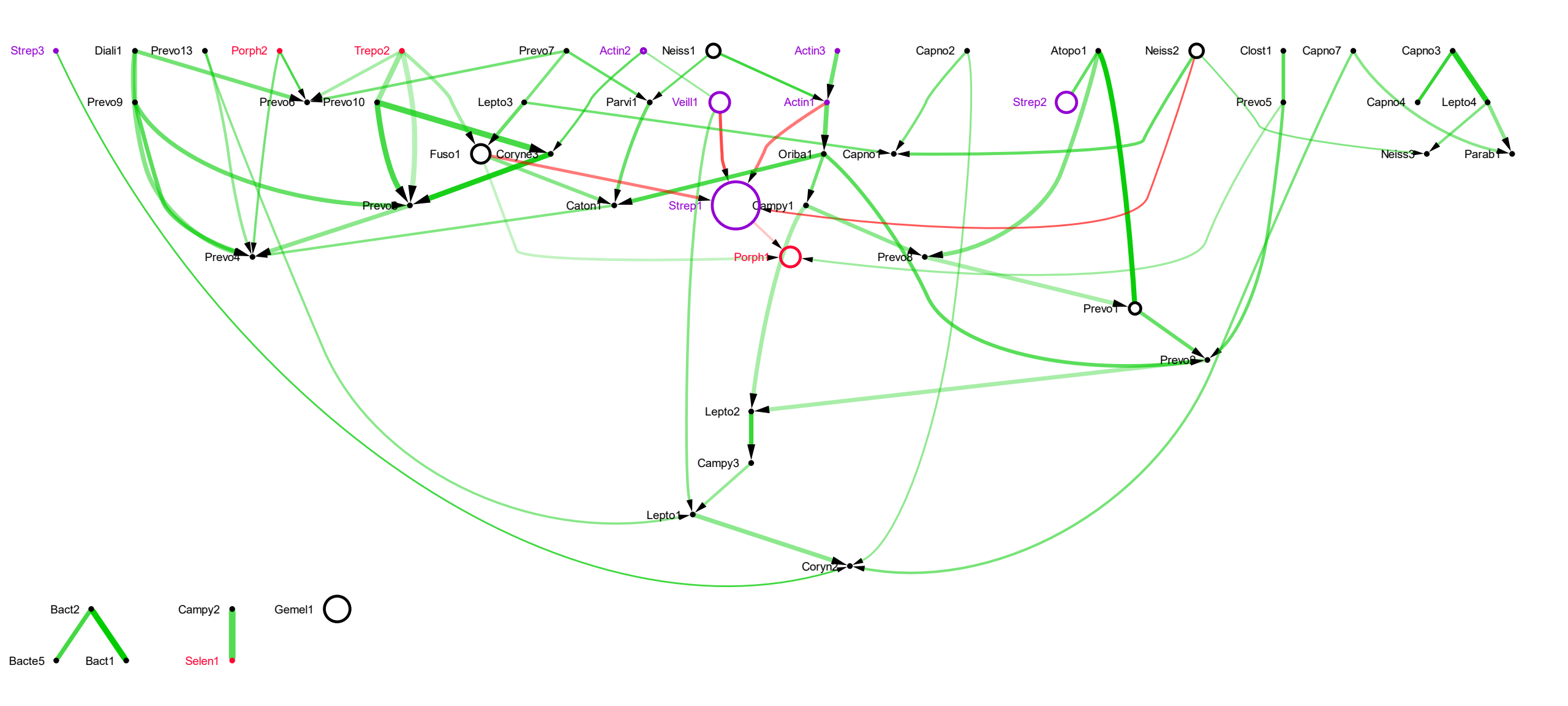

Supplement: Supplementary file 1 — Additional file 1 sBN of Buccal mucosa [file 12864_2020_7065_MOESM1_ESM.pdf]

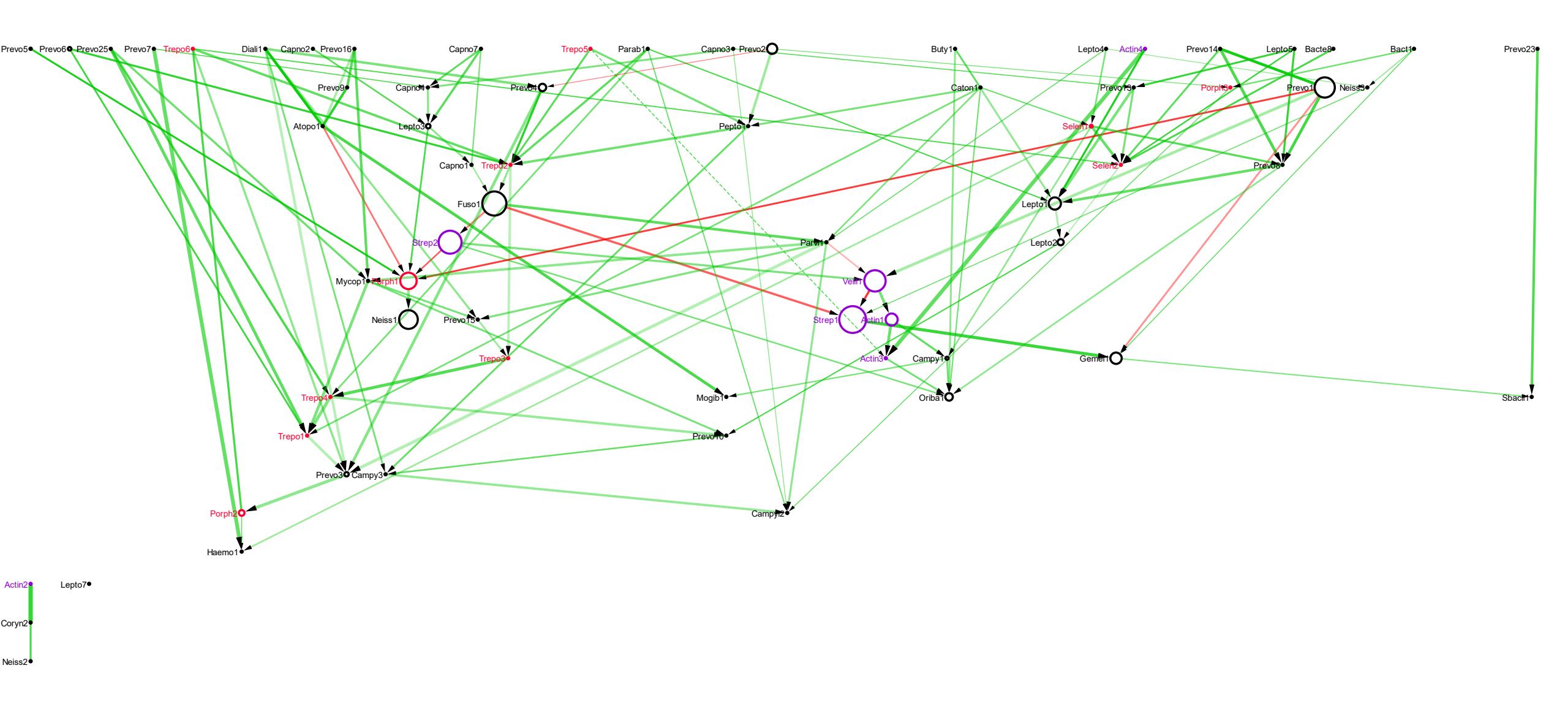

Supplement: Supplementary file 2 — Additional file 2 sBN of Palatine tonsils [file 12864_2020_7065_MOESM2_ESM.pdf]

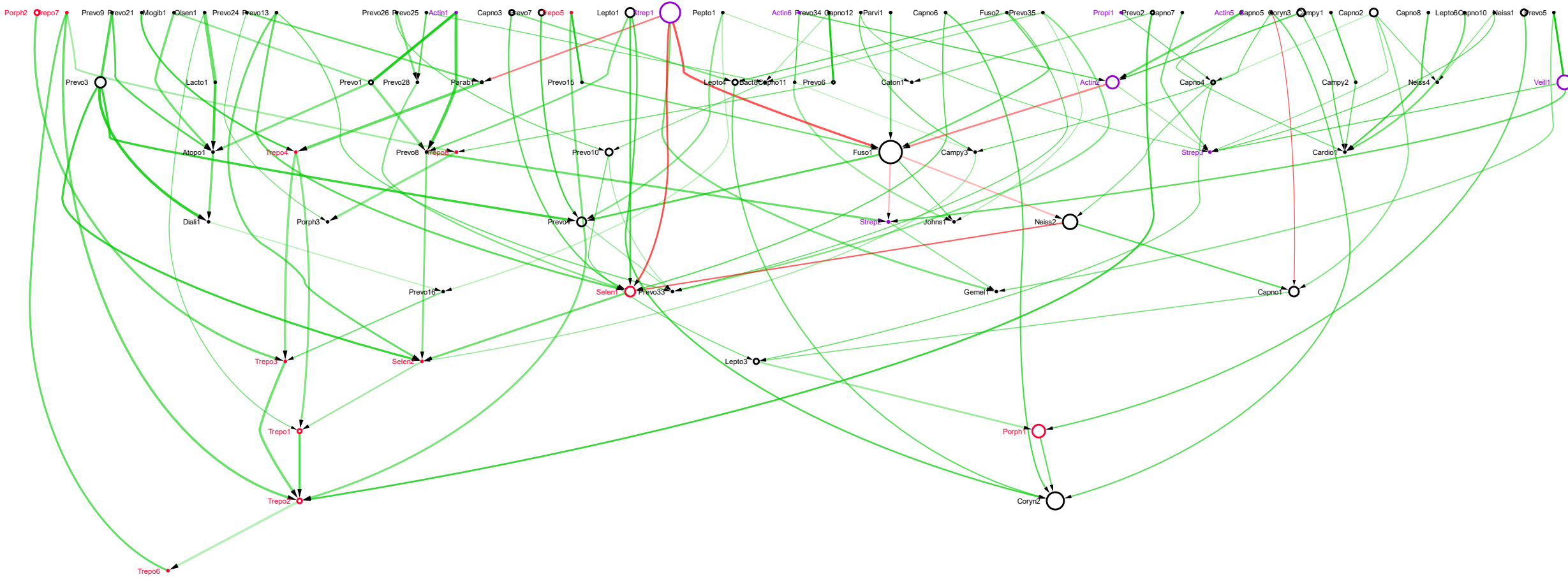

Supplement: Supplementary file 4 — Additional file 4 sBN of Subgingival plaque [file 12864_2020_7065_MOESM4_ESM.pdf]

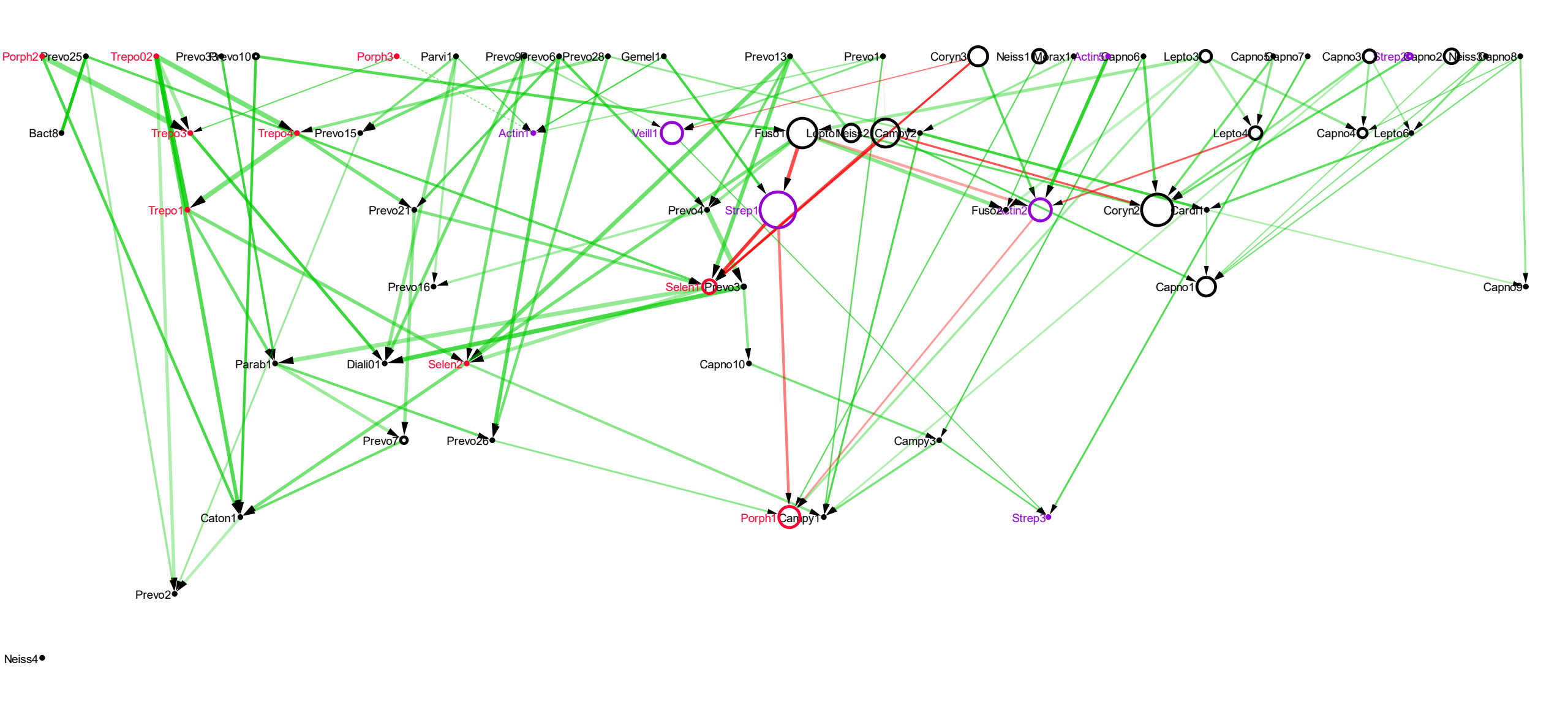

Supplement: Supplementary file 5 — Additional file 5 sBN of Supragingival plaque [file 12864_2020_7065_MOESM5_ESM.pdf]

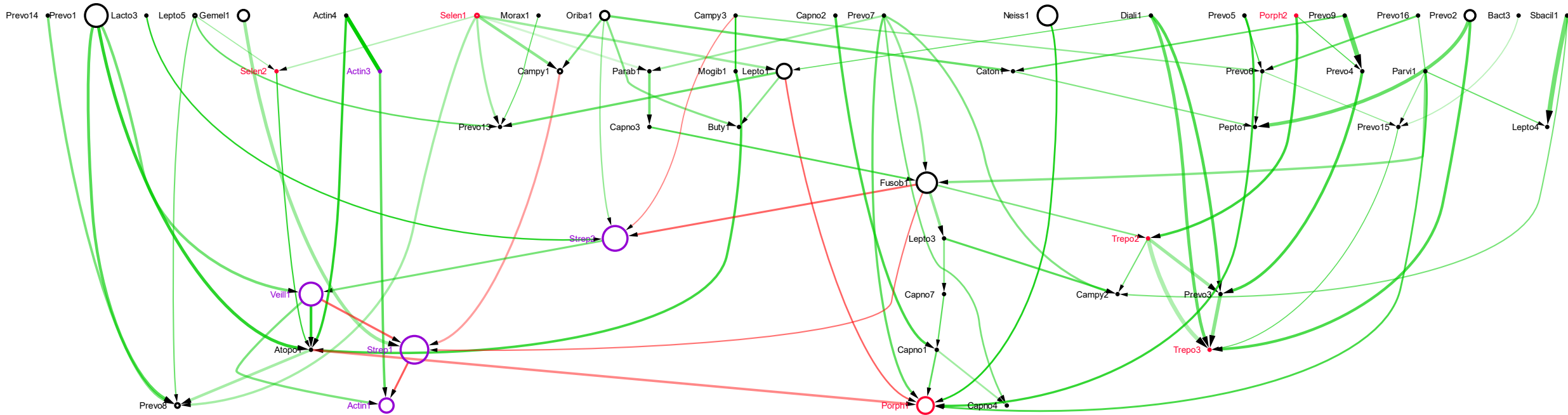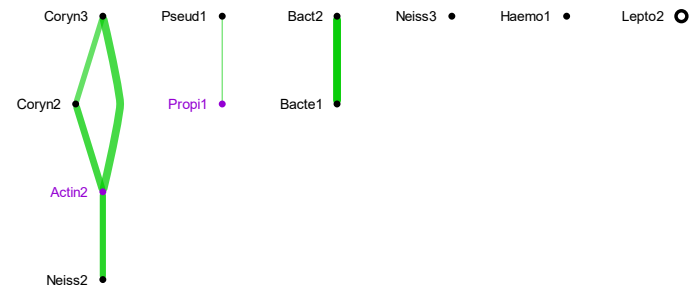

Supplement: Supplementary file 6 — Additional file 6 sBN of Throat [file 12864_2020_7065_MOESM6_ESM.pdf]

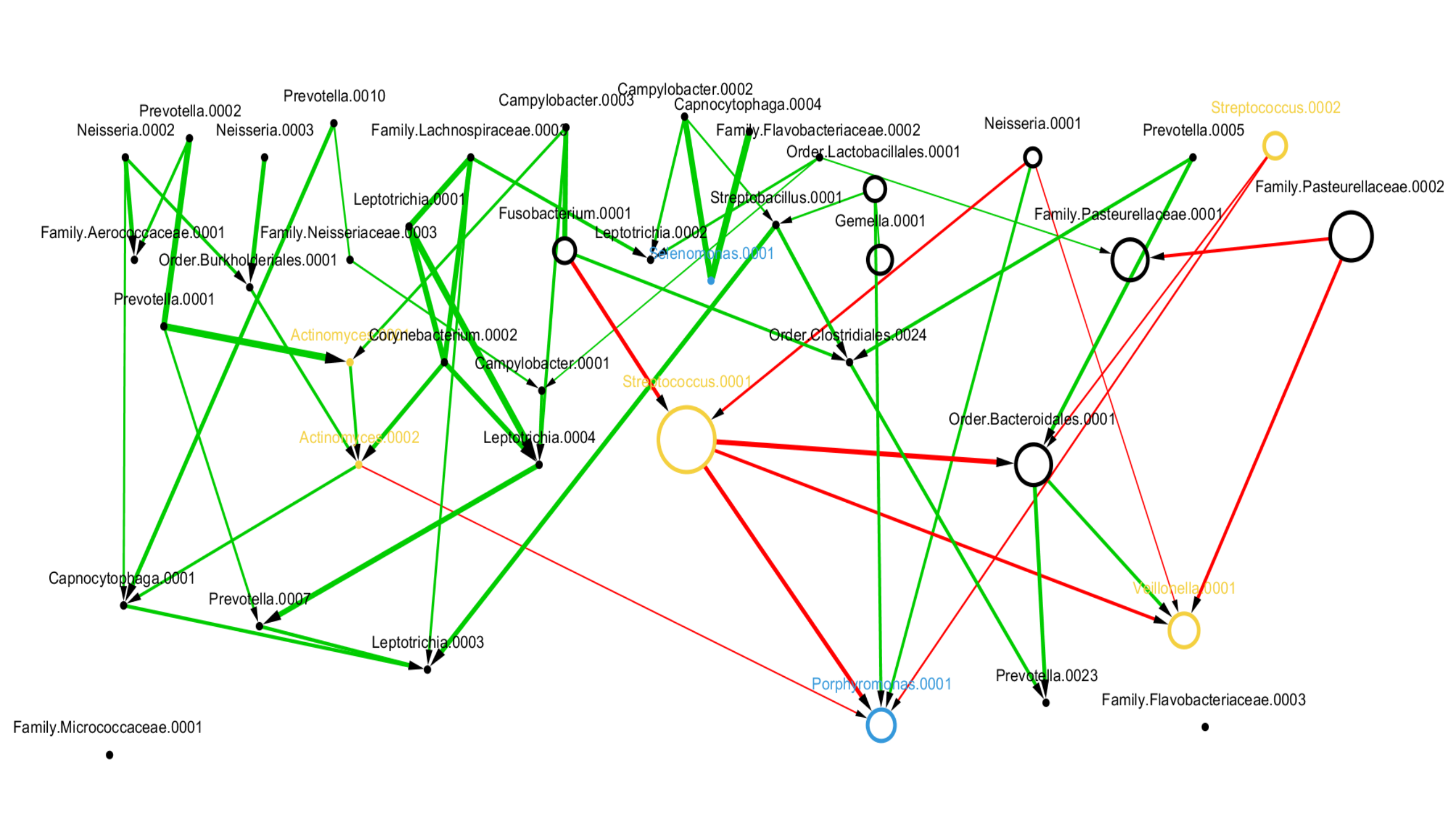

Supplement: Supplementary file 7 — Additional file 7 sBN of Keratinized Gingiva with 99.99% taxa [file 12864_2020_7065_MOESM7_ESM.pdf]
