# Supplementary material for: Inferring directional relationships in microbial communities using signed Bayesian networks
Source: BMC Genomics. 2020 Dec 21;21(Suppl 6):663. doi: 10.1186/s12864-020-07065-0 (PMC7751116; doi:10.1186/s12864-020-07065-0)
Supplement: Supplementary file 3 — Additional file 3 sBN of Saliva [file 12864_2020_7065_MOESM3_ESM.pdf]

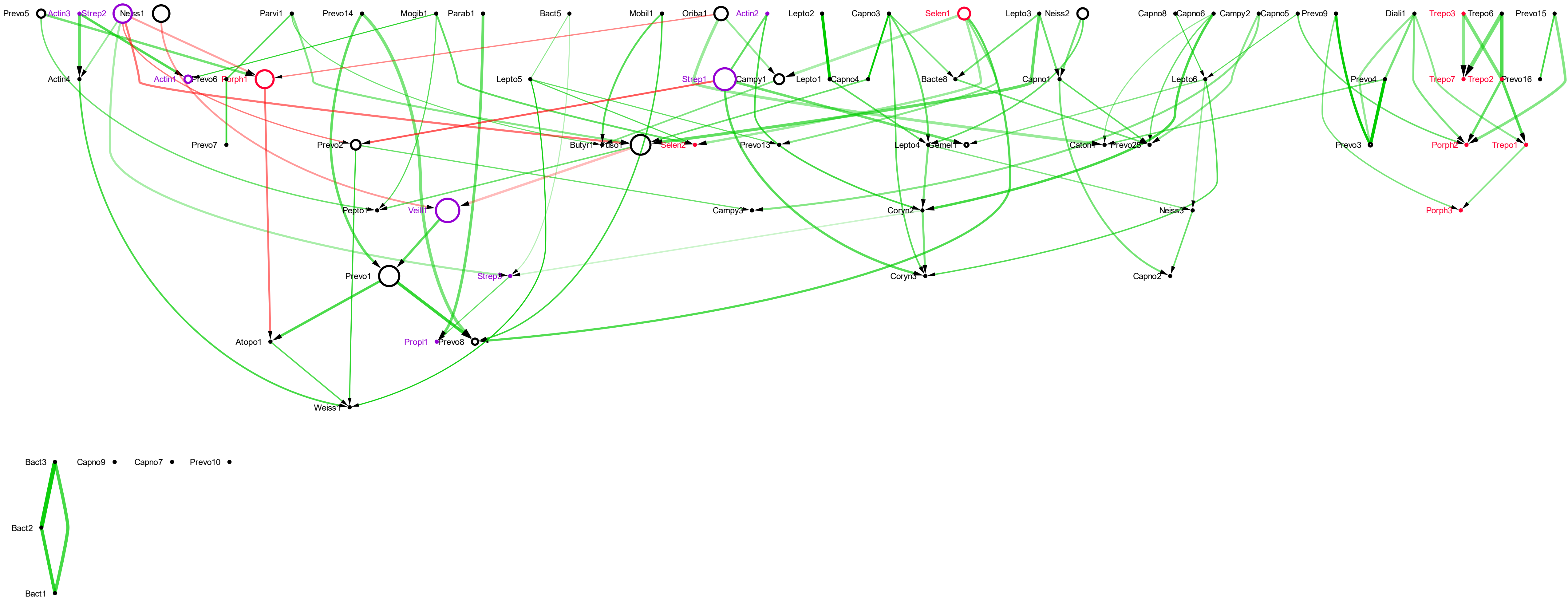

Prevo5
Actin3
Strep2
Neiss1
Parvi1
Prevo14
Mogib1
Parab1
Bact5
Mobil1
Oriba1
Actin2
Lepto2
Capno3
Selen1
Lepto3
Neiss2
Capno8
Capno6
Campy2
Capno5
Prevo9
Diali1
Trepo3
Trepo6
Prevo15
Actin4
Actin1
Prevo6
Porph1
Lepto5
Strep1
Campy1
Lepto1
Capno4
Bacte8
Capno1
Lepto6
Prevo4
Trepo7
Trepo2
Prevo16
Prevo7
Prevo2
Butyr1
Fuso1
Selen2
Prevo13
Lepto4
Gemel1
Caton1
Prevo25
Prevo3
Porph2
Trepo1
Pepto1
Veil1
Campy3
Coryn2
Neiss3
Porph3
Prevo1
Strep3
Coryn3
Capno2
Atopo1
Propi1
Prevo8
Weiss1
Bact3
Capno9
Capno7
Prevo10
Bact2
Bact1
